# Video-game-assisted physiotherapeutic scoliosis-specific exercises for idiopathic scoliosis: case series and introduction of a new tool to increase motivation and precision of exercise performance

**DOI:** 10.1186/s13013-016-0104-9

**Published:** 2016-11-24

**Authors:** Christine Wibmer, Petra Groebl, Alexander Nischelwitzer, Beate Salchinger, Matthias Sperl, Helmut Wegmann, Hans-Peter Holzer, Vinay Saraph

**Affiliations:** 1 Department of Orthopedic Surgery, Medical University of Graz, Auenbruggerplatz 5, A-8036 Graz, Austria; 2 University of Applied Sciences, Alte Poststraße 149, A-8020 Graz, Austria; 3 Pediatric Orthopedic Unit, Department of Pediatric Surgery, Medical University of Graz, Auenbruggerplatz 34, A-8036 Graz, Austria; 4 Institute of Sport Science, University of Graz, Mozartgasse 14, A-8010 Graz, Austria

**Keywords:** Scoliosis, Exercise therapy, Motivation, Video games

## Abstract

**Background:**

It is important to monitor how patients with juvenile and adolescent idiopathic scoliosis comply with their physiotherapeutic scoliosis-specific exercises (PSSE). Physiogame, a newly developed video game using the Game-Trak 3D interactive game controller, combines correct PSSE performance with gaming. It tracks the position of the working limb in 3D space during the exercises as participants aim to hit certain targets and avoid others, and gives direct feedback by stopping the game if the working limb leaves the target 3D space, which is chosen to secure the corrective position according to the Schroth method. Physiogame records the quality and frequency of the exercises performed. We aimed to investigate the influence of this tool on motivation to perform regularly and, correctly, and with self-assessment of performance quality.

**Methods:**

This case series included 8 consecutive patients with idiopathic scoliosis (thoracolumbar 7, lumbar 1), ages 7-13 years, all female and treated according to SOSORT guidelines; the COBB angle of primary curve at the start of brace therapy was 22-34°. In addition to Full Time Rigid Bracing (FTRB, Cheneau) and PSSE (Schroth), the participants were to perform two standardized Schroth exercises (muscle cylinder in standing position, mainly addressing the thoracic curve, and in side-lying position, mainly addressing the lumbar curve) with video game assistance every day for 6 months. The development (first to last month) of the following parameters was analyzed with descriptive methods: the actual training time to assess motivation, the ratio of the actual playing time versus total playing time to assess exercise improvement, and self-assessment of quality of performance.

**Results:**

The average number of sessions with Physiogame was 217 per study participant (range 24 to 572, the study protocol targeted at least 180); actual training time decreased from 79 to 52 min (first to last month). Actual playing time increased from 73% of the total playing time to 83% (first to last month), and positive hits per second from 0.33 to 0.56. Self-assessment increased from “good” to “very good”. The curve angles (°Cobb) were maintained over the study period (upper thoracic mean -1.3°, median -1°; lower thoracic mean 3°, median 2°; lumbar mean 0.5, median 0).

**Conclusions:**

The improvement we saw in exercise performance, is thought to result primarily from the direct given feedback during the game, as the exercises themselves were already familiar to the patients. The synchronous recording of actual training time allows evaluation of Schroth therapy for idiopathic scoliosis, since both prescribed training time and actual training time are captured. No comparable tool was found in literature.

**Electronic supplementary material:**

The online version of this article (doi:10.1186/s13013-016-0104-9) contains supplementary material, which is available to authorized users.

## Background

For the treatment of juvenile and adolescent idiopathic scoliosis, the SOSORT guidelines [1] recommend stage specific treatment strategies. Conservative treatment consisting of Full Time Rigid Bracing (FTRB) and physiotherapeutic scoliosis-specific exercises (PSSE) [2] is recommended in cases of curve angles between 20° and 40° Cobb and/or curve progression in skeletally immature patients. Various types of PSSE and FTRB are equally advisable according to the SOSORT recommendations including the Schroth method [3] as PSSE and the Cheneau brace as FTRB, which we use in our department. The literature review underlying the SOSORT guidelines also revealed a need for research strategies to evaluate conservative treatment of idiopathic scoliosis (CTIS) [1]. Even though several reports have shown the positive influence of PSSE on curve progression and quality of life, the difficulty of monitoring compliance and correct performance of PSSE limits clinical research on the effect of PSSE [4–6]. Recent reports confirm the importance of supervised PSSE [7, 8], but intensive supervision by a physiotherapist is not feasible in an outpatient setting, where the patients have to practice on their own between their weekly supervised physiotherapy sessions.

Exergames are defined as virtual reality video games that provide physical exercise, and have been described and validated as useful tools in neurologic rehabilitation [9–11] and in the treatment of overweight children [12, 13], among others. However, we could find no comparable reports analyzing exergames in the treatment of idiopathic scoliosis.

The concept of the Schroth method is to correct the deformity in three dimensions using repeated asymmetrical 3D spinal correction exercises. They aim to straighten the scoliosis by corrective positioning, elongation of the spine, de-rotation the rib cage and pelvis, side shift of the thorax, and shoulder corrections in standing, sitting and lying positions. The patients first adopt a specific straightened position, with misaligned and rotated pelvis and shoulders held in an over-corrected position; then the patients use a corrective breathing technique (Schroth rotational breathing/rotational angular breathing - RAB), breathing into the collapsed areas of the rib cage. Breathing in this manner aims at decreasing the rotation in both the rib cage and spine, and improving lung capacity and rib mobilization. The arm position is important to correct the rib cage and shoulder in the standing position; a rice bag passively corrects the lumbar curve in the side-lying position and achieves the desired pelvic alignment [14]. These auto-corrective exercises provide proprioceptive training, enhance muscle endurance [7], and are intended to have a stabilizing effect on the vertebral column. The main long-term goal of PSSE is a stretched and straightened posture of the spinal column, not only in the training setting but in everyday activities as well [2]. The motor learning process needed to change a movement pattern so completely comprises three stages [15, 16]: the cognitive phase (understanding a new movement), the associative phase (refinement of the newly acquired movement), and the autonomic phase (movement can be accomplished almost automatically). A study on the feedback demand of young children whose motivation and learning progress are to be improved reported that more feedback leads to more precise movements [17]. This is in contrast to adults, in whom less feedback leads to higher precision [17]. If children can choose the extent of feedback for themselves, they request it far more often than adults [18], and their motor learning curve rises more steeply with higher feedback frequency [18]. Huber [19] described the advantages of giving feedback during the exercises exactly at the moment when the movement starts to deteriorate, not later, not earlier. Based on these insights, a video exergame (combining game and exercise) was designed to help to motivate young patients with idiopathic scoliosis to perform their home exercises regularly and to adjust their posture during the exercises. Distracting the mind with the game during the exercise session could also hasten progress to the autonomic phase of motor learning [17]. The recorded training data further allow a more realistic assessment of the PSSE method (in our case, Schroth).

This Physiogame, developed by the IT department of the FH JOANNEUM, University of Applied Sciences, Graz, Austria, for this study, combines PSSE with a shooting game [9]. The 3D positions of the trunk and extremity are individually adjusted to the corrective posture desired; the game only continues when the posture is correct. Motivation to perform the exercises regularly and correctly is hypothesized to be enhanced by the gaming setting and the real-time-feedback; to confirm these hypotheses, we designed an original study to determine whether an exergame could improve motivation and correct performance of the specific exercises prescribed to treat juvenile and adolescent idiopathic scoliosis.

Our research questions were as follows: how does the implementation of video game assisted PSSE help young patients carry out their exercises in terms of motivation to perform their PSSE steadily and correctly, better performance of the exercises and self-assessment of quality of performance (stabilization of the vertebral column)?

## Methods

### Design and setting

This study was designed as a prospective case series and was approved by the local ethics committee (EK-Nr. 23-235 ex 10/11). It was performed at the outpatient department of a tertiary center specialized in conservative and surgical treatment of scoliosis.

### Subjects

After written informed consent was obtained, 8 consecutive patients with idiopathic scoliosis eligible for this study were included prospectively. Inclusion criteria were treatment of juvenile or adolescent idiopathic scoliosis following the SOSORT treatment recommendations [1], including FTRB and PSSE. The inclusion criteria did not restrict curve type or gender. Exclusion criteria were scoliosis other than idiopathic, neurological disorders and cognitive limitations of the patients.

### Assessment

Primary outcomes of this study were the results of the three research questions: (1) motivation to perform their PSSE steadily and correctly, (2) increase in correct performance of the exercises, and (3) self-assessment of quality of performance. Secondary outcomes were change in curve severity and course of disease.

The course of the disease was assessed at three-month intervals by an orthopedic surgeon specialized in spinal deformities, with medical history (menstrual status, medication, present complaints), inspection of the back in stance and with Adam’s forward bending test (rotation, flexibility, waist and shoulder asymmetry), and measurement of height and weight. At six-month intervals a plain radiograph of the entire vertebral column was taken with the brace in place. The brace is worn when an x-ray is made to comply with the radiologic standards of our pediatric department to minimize radiation burden during the years of brace treatment and to assess the in-brace-correction regularly. For this study the Cobb angles were measured manually and blinded by one specialized orthopedic surgeon. Acknowledging the known intra-observer variability of approximately 5° upon manual measurement [20, 21], the analysis of the change of curve severity was considered constant if it was less than 5°.

The Schroth curve types were assessed according to the original Schroth classification [3, 22].

### Intervention

Once included in the study, for six months the patients augmented their prescribed exercises with an individualized home training program including the Physiogame, and were instructed in keeping a training diary. The home training program (duration approximately 30 min) was to be performed throughout the study period and at least 5 times a week. It comprised two standardized Schroth exercises using Physiogame (for approximately 10 min, which included assuming the corrective positions for the two muscle cylinder exercises and playing the game in that posture), individualized exercises for elongation and auto-correction, and exercises in front of a mirror to transfer the corrective posture to everyday activities (for approximately 10 min). Finally, there were two Schroth breathing exercises (RAB, for approximately 10 min), which were chosen for the patients individually according to their specific deformities. Three physiotherapists specially trained in the Schroth technique taught them both the specific sequence of exercises for the home training program and the two Schroth exercises as standardized for performance with Physiogame [3, 14]. They also supervised the correct performance of the Schroth exercises in the outpatient PSSE sessions throughout the study period. All patients had such supervised PSSE sessions (duration: 45 min) weekly for the first seven weeks, and then every two weeks for the rest of the study period. In each physiotherapy session the prescribed Schroth exercises were practiced under supervision, including the two standardized muscle cylinder exercises with Physiogame, the individualized exercises and the two individual RAB breathing exercises (approximately 15 min). Another 10 min each were devoted to mobilizing massages, soft-tissue and fascia techniques and transfer of the corrective posture and RAB into everyday activities.

The two standardized Schroth exercises [3, 14] were chosen mainly to address the lumbar curve with the “muscle cylinder in side-lying position” and the thoracic curve with the “muscle cylinder in standing position,” with a working limb free to play the video game. The corrective postures were standardized as follows:

1. “Muscle cylinder in side-lying position”: the patient has to be in a corrective or over-corrective position to open the concave side of the lumbar curve and allow training with a straightened spine. To this end, a rice pad is placed under the downward pointing convex side of the lumbar curve. The upper extremity on the concave side of the thoracic curve is straightened and raised overhead (shoulder flexion); another rice pad is put under the shoulder to straighten the thoracic curve. The opposite arm is positioned in front of the chest with flexed elbow to allow the scapula to be pushed against the rib hump during the exercise. The lower extremity lies on the floor with hip and knee flexed (90° flexion). The pelvis is held strictly perpendicular. The working limb is the leg on the concave side of the lumbar curve, which rests on a cuboid in stretched position. To begin the muscle exercise the patient abducts this leg, thereby derotating the lumbar spine and strengthening the muscles on the concave side of the lumbar curve, which are in an optimal straightened corrective posture (Fig. 1).

**Fig. 1:**
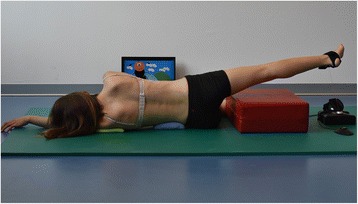
Patient positioning for the muscle cylinder in side-lying position with Physiogame sensor cuff attached to the right ankle (left convex lumbar curve)
2. “Muscle cylinder in standing position”: the intention of the corrective posture lies in stretching the contracted concave side of the thoracic curve and rib cage for the RAB. The lower extremity on the concave side of the lumbar curve is the support leg; the other leg is stretched and abducted, and rests on a low stool. The hand on the convex side of the thoracic curve lies on the hip to push the scapula against the rib hump during the exercise. The arm on the concave side of the thoracic curve is the working limb, which is stretched upward to form a straight line with the abducted opposite leg, thereby also contracting the abdominal muscles on the convex lumbar side. The RAB directs the inspiration into the thoracic concavity; exhaling in this posture contracts the ribcage on the convex side. Coordination for this exercise is demanding: the derotation of the thoracic curve through RAB is amended by the derotation of the lumbar curve using the same principles as in the side-lying exercise (Fig. 2).

**Fig. 2:**
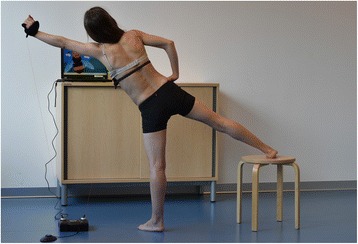
Patient positioning for the muscle cylinder in standing position with Physiogame sensor cuffs attached to left wrist and the bra band (right convex thoracic curve)

**Fig. 3:**
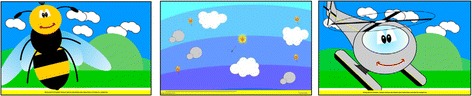
Physiogame, three different game environments

The Physiogame software was specifically designed for use with these two standardized exercises. It uses the hardware of the Game-Trak 3D interactive game controller (In2Games Ltd, Rickmansworth, UK), a 3-dimensional game control system based on position tracking. This hardware was selected on the basis of its use in several commercial applications of video games. The Game-Trak comprises a base unit connected to a personal computer and two cuffs to track the exact position of two points at one time. One is placed on the extremity in training (wrist for the “muscle cylinder in standing position” or ankle for the “muscle cylinder in side-lying position”); the other sensor is placed on the trunk (attached to the bra or a girdle) to monitor the stabilization of the spinal column in the muscle cylinder in standing position (for the muscle cylinder in side-lying position the second sensor is on stand-by, as the patient’s trunk is stable, positioned side-lying on the floor). Three different game environments are offered: “catching flowers”, “prick balloons with a needle” and “landing the helicopter” (Fig. 3). In each game there are positive targets to hit and negative targets to avoid. At the same time the working limb should not leave a predefined 3-dimensional space in order to retain the corrective posture during the exercise. The 3D space is adjusted individually for each patient to assure correct positioning during the game. The selection of the working limb depends on the Schroth curve type. The duration of one game can be adjusted to different levels of difficulty (i.e. by adjusting the duration to patient’s level of muscle endurance to maintain the corrective posture). The game stops the time count if the extremity leaves the predefined space. This results in a gross or total playing time and a net or actual playing time during which the working limb is inside the 3D target space (actual training time). As soon as one level has been completed, a game report appears with the duration of the game, score, positive and negative target hits, positive targets hit per second as well as a query concerning self-assessment: “today I did well” (range 1 = excellent to 5 = poor), “today I managed to stabilize my vertebral column” (range 1-5), and “I want to play again/quit playing” as the last question (Additional file 1).

### Statistical analysis

The data collected via the video game were analyzed with descriptive methods.

The duration of the study was 6 months; the data from first and last month of the study period were compared to analyze changes in motivation, correct exercise performance and self-assessment. The total amount of actual training time per study participant was recorded and together with the comparison of net playing time in the last and first months, formed the parameter for motivation. The changes in the actual playing time per game and positive hits per second over the study period were calculated to analyze performance improvement. The development in self-assessment (today I did well, today I managed to stabilize my vertebral column) was analyzed to evaluate changes in the quality of performance as perceived by the patient.

## Results

All study participants were female. At the beginning of the study the patients’ average age was 10 years (range7 to 13 years), and they had worn a brace and performed accompanying PSSE for an average of two years (minimum 9 months, maximum 4 years) before enrollment in the study. The localization of the primary curve was thoracolumbar in seven cases and lumbar in one. The Schroth curve types were 3c in three cases, 3cp in two and 4c in three cases. The Cobb angles of the primary curve at the start of the study ranged between 16° and 26° (with applied brace). The curve severity did not change significantly over the study period. On average, the curve angle decreased by 1° Cobb (median 1°) in the upper thoracic curve and increased by 3° Cobb (median 2°) in the lower thoracic curve and 0.5° Cobb (median 0°) in the lumbar curve (with applied brace) (Table 1). Table 1 also shows details of the long-term data from the patient collective: at the start of brace therapy, the severity of the primary curves was 22-34° Cobb (without brace), the mean total duration of brace therapy was 4 years, mean growth during brace therapy was 27 cm. After brace weaning the curve severity ranged from 18-36° Cobb in the primary curve. No patient had to undergo surgery during or after this conservative treatment.

**Table 1:** Characteristics of 8 study participants with idiopathic scoliosis

ID-No.		1	2	3	5	6	7	8	9	Mean	Median
Gender		f	f	f	f	f	f	f	f		
Age at start of bracing [years.months]		9.3	5.11	6.7	6.6	12.4	12.5	13.5	11.8	9.9	10.6
Age at start of study [years.months]		13.2	10.2	7.8	10.6	13.11	13.10	13.11	13.10	10.7	13.6
Duration of bracing [years.months]		5.2	7.10	5.7	7.6	2.8	1.8	2.7	2.5	4.5	3.11
Increase in body height during bracing [cm]		34	40	38	50	18	12	10	14	27	26
Curve at start of bracing[°Cobb]	upper thoracic	18	4	22	22	n.a.	n.a.	10	16	15	17
	lower thoracic/thoracolumbar	24	30	24	34	14	24	22	26	25	24
	lumbar	16	22	16	18	34	32	20	22	23	21
Curve at end of bracing [°Cobb]	upper thoracic	26	8	30	44	n.a.	n.a.	8	14	22	20
	lower thoracic/thoracolumbar	28	20	30	36	12	20	26	18	24	23
	lumbar	24	12	12	30	24	20	24	10	20	22
Curve at start of study [°Cobb]^a^	upper thoracic	26	8	22	20	n.a.	n.a.	8	18		
	lower thoracic/thoracolumbar	10	16	26	10	16	16	20	18		
	lumbar	12	16	26	10	20	14	20	20		
Curve at end of study [°Cobb]^a^	upper thoracic	22	10	20	14	n.a.	n.a.	8	20		
	lower thoracic/thoracolumbar	22	20	26	18	20	16	20	14		
	lumbar	26	14	22	12	22	20	16	10		
Change of curve severity during the study [°Cobb]^a^	upper thoracic	-4	2	-2	-6	n.a.	n.a.	0	2	-1.3	-1
	lower thoracic/thoracolumbar	12	4	0	8	4	0	0	-4	3	2
	lumbar	14	-2	-4	2	2	6	-4	-10	0.5	0
Schroth curve type		4c	3c	3cp	3cp	4c	3c	4c	3c		
		r	r	r	r	l	l	l	r		

*f* female, *n.a.* not applicable, *r* right, *l* left, ^a^measured on radiograph with applied brace

### Motivation

As the participants’ training diaries were too incomplete to provide data for analysis, we had to rely on the Physiogame data. The average number of gaming sessions during the six months of the study was 217, but the range was very broad, with a minimum of 24 and a maximum of 572 gaming sessions in six months, while the study protocol called for training sessions at least five times a week and at least 180 sessions in total (Table 2). The playing time per game averaged 72 s (gross playing time) and 56 s (net playing time); the inter-individual differences reflect the individual difficulty adjustments. The patients used the Physiogame tool for an average of 79 min during the first month and 52 min during the last month (net playing time). This net playing time only covers active play; the training session, however, includes the time the patients needed to assume the corrective posture and then to start the video game. The numbers of training sessions are thus a more realistic representation of the amount of time spent performing Schroth therapy than the gaming time alone. More detailed analysis revealed major differences in training habits: some patients chose to train several times a day followed by several days’ break, while others trained every day. Furthermore, some patients chose to perform mainly in one training position (e.g. patient No.2) did not perform a single session in the standing position but completed 261 training sessions in the side-lying position.

**Table 2:** Results of 8 study patients using Physiogame over a period of 6 months

ID No.		1	2	3	5	6	7	8	9	Mean	Median
Total number of game sessions		83	261	64	361	139	572	233	24	217.1	186.0
Whereof in standing position		42	0	60	149	139	3	119	24	67.0	51.0
Whereof in side-lying position		41	261	4	212	0	569	114	0	150.1	77.5
Net playing time [min]	first month	85	50	10	132	17	94	101	1	61.1	67.7
	last month	15	34	17	65	10	128	88	4	45.7	25.7
	ratio last to first									111.2	
Playing time per game [sec]	mean gross	63.8	71.4	82.8	83.3	61.1	58.3	91.9	66.2	72.4	68.8
	mean net	60.0	58.9	45.0	66.8	40.1	52.9	75.1	46.9	55.7	55.9
Net playing time [%]	mean	94.1	82.5	54.4	80.2	65.7	90.7	81.7	70.8	77.0	81.0
Net playing time/gross playing time [%]	first month	94.4	77.6	48.3	74.7	40.7	85.8	80.4	80.2	72.8	78.9
	last month	93.5	85.8	64.2	85.7	91.3	91.7	85.0	69.6	83.4	85.8
	ratio last to first	99.0	110.6	132.8	114.9	224.5	107.0	105.7	86.8	114.6	108.8
Positive hits per second	first month	0.64	0.34	0.13	0.22	0.17	0.57	0.46	0.14	0.33	0.3
	last month	0.86	0.68	0.17	0.55	0.53	0.76	0.68	0.21	0.56	0.6
	ratio last to first [%]	135.4	202.9	133.8	249.1	309.1	133.4	146.6	144.8	166.5	145.7
Mistakes >80% in direction (x,y,z)		x	y	y	y	y	x	y	y	y	
Self-assessment: today I did well [1(excellent) - 5(poor)]	mean	2.5	1.6	2.4	2.0	2.5	2.6	2.4	2.2	2.3	2.4
	first month	2.4	2.0	3.2	2.1	2.8	2.6	2.6	4.0	2.7	2.6
	last month	1.9	1.3	2.1	2.0	2.5	2.6	2.1	3.5	2.3	2.1
Self-assessment: today I managed to stabilize my vertebral column [1–5]	mean	2.2	1.7	2.3	2.1	2.5	2.4	3.0	2.3	2.3	2.3
	first month	2.2	2.0	2.7	2.2	3.1	2.5	2.9	3.0	2.6	2.6
	last month	1.6	1.4	1.8	2.0	2.9	2.3	2.8	3.5	2.3	2.2

Gross playing time: total time, including stops to correct posture

Net playing time: playing time with working limb inside the target 3D space

### Increase in correct performance of the exercises

On average the study participants had an actual training time of 77% of the gross playing time (range 54–94%). In the first month they managed to stay in the predefined 3D space 73% of the gross playing time per game (range 41–95%). In the last month of the observation period this increased to 83% (range 63–94%). The children improved their performance of the exercise on average by 15% (range from a decrease of 13% to an increase of 125%) (Table 2). Even though the training habits differed inter-individually, the average results on increase in correct exercise performance are consistent, when the results of improvement per game are compared with the average increase of net playing time from first to last month of 11% (Table 2). The improvement in staying in the corrective posture autonomously and being able to focus more on the game is reflected in the average increase of positive hits per second: they increased from 0.33 in the first month to 0.56 in the last month, for an average increase of 66%.

Regarding the predefined 3D space in which the extremity was to be kept, six out of eight children made the most mistakes in positioning the arm/leg in the forward dimension, toward the screen. This is the third dimension, which is not reproduced on the two-dimensional screen. Two study participants had a pronounced tendency to let their arm/leg droop (see column “mistakes >80% in direction x,y,z”, Table 2).

### Self-assessment of quality of performance

The self-assessment of general performance (“today I did well”) stayed almost the same over the study period, with an average of 2.7 (good) in the first month and 2.3 (very good) in the last month. Similarly, self-assessed stabilization of the vertebral column changed only slightly from 2.6 (good) in the first month to 2.3 (very good) in the last month.

## Discussion

In this study, we added video game assistance to PSSE in the treatment regimens of growing children with idiopathic scoliosis. The treatment consisted of FTRB and PSSE, and the Physiogame tool was integrated into two specific exercises that were added to a regular home Schroth program. The intention was to increase the children’s motivation to adhere to their prescribed training program, to encourage them to perform their exercises correctly, and to increase their capacity to assess for themselves their ability to stabilize the spine.

As to motivation to perform the exercises regularly (i.e. daily as prescribed or at least five times a week), we saw a tendency to neglect the video game toward the end of the study period, suggesting that by six months, the game has become boring, especially since the level of difficulty was never adjusted. This could be addressed outside this study protocol by using the game only for a shorter period, or by adjusting the level of difficulty. Our everyday experience indicates that the playful diversion Physiogame provides may help to motivate the children to perform the tiring muscle cylinder exercises if they have already been doing them routinely.

This tool motivated the participants to perform their exercises correctly and their precision increased throughout the study. The recorded data from Physiogame clearly indicated that the patients’ actual training habits differ from what was prescribed, at least as far as that part of the exercise session is concerned. Due to the very incomplete training diaries, we have no data with which to evaluate the rest of the prescribed PSSE. This is in accordance with recent reports [7, 8], that the findings for prescribed but unsupervised PSSE are to be interpreted with caution. As the patients already knew, the Schroth exercises (muscle cylinder in standing and in side-lying position), the improvement seen in their correct performance may accrue from the real-time feedback that helps them to keep their corrective posture as they play the game. This is comparable to the trainer-trainee situation in competitive sports, where the athlete’s performance during training sessions is also corrected in real time. Physiogame so can serve as a sort of “take-home-therapist” to keep the children on the track when they are practicing. We found no reports on whether children benefit more if feedback is reduced over a longer training period, as such studies as are available address only short-term learning effects with uncomplicated movements [17]. Besides the immediate feedback, by getting the child’s mind off the exercise, the game can facilitate progress toward the autonomic phase of motor learning. Video games are designed to encourage the player to perform well to get a good score, and to improve that score by playing another round. This may also improve motivation to perform the exercises regularly and correctly in every session.

Interestingly, this tool does not seem useful for self-assessment as there was only a weak correlation between positive hits per seconds and positive self-evaluation. To clarify this, we questioned the participants directly. Their answers indicated that they tended to think in terms of only the last three sessions, rather than the whole time span of the study from start to finish.

We acknowledge that the study had some limitations. The number of study participants was too small to allow a statistical analysis. A comparison to a control group was not possible as no comparable data on training intensity could be gained in this setting in which a video game records the training data and without the game, no objective data could be generated by a control group. That the prospectively recruited study population consisted of girls only to some extent reflects the gender distribution of idiopathic scoliosis and the small number of children who present with the disease. The game design is two dimensional, but exercises have to be performed correctly in three dimensions, which is challenging for young children. This could be addressed, of course, by developing a 3-dimensional game. The game was initially designed for the German-speaking world, which restricted its broad use, but it is now available in Italian and English as well. The game design addresses young children, who respond better to the permanent, real-time feedback than older patients who are better motivated with less feedback; for them other methods should be developed to respond to their needs.

The literature covers two applications of exergames: commercial video games designed to improve fitness in general, and therapy-specific games, designed to aid in the performance of disease-specific exercises. Commercial video games can be used as therapy-assistance and are widely available [9, 13]. Their availability supports their use as therapy-assistance even though they do not address disease-specific exercises. Therapy-specific games on the other hand show clinical promise in supporting physiotherapeutic goals, but still need to be backed up by randomized controlled trials that show solid evidence of their effectiveness [9, 11].

## Conclusions

We present a video game designed especially for PSSE (“Physiogame”), which addresses the feedback demand of children during the exercise session (“take home therapist”) [17] and provides playful diversion during the performance of otherwise dreary exercises. No comparable tool for PSSE could be found in literature. The correct performance of the exercises improved during our study, even though the participants were already familiar with the exercises, but only larger studies will allow statistical analysis. Physiogame could be suitable in such future studies to evaluate the impact of PSSE in general and the Schroth method in particular. Documentation of the gross and net training times would allow more objective evaluation of the effect of PSSE on the management of idiopathic scoliosis in children.

## Additional file

Additional file 1: Video of patient playing Physiogame in the side-lying position. (right convex thoracic and left convex lumbar curve). (MP4 9716 kb)

## Abbreviations

CTIS: Conservative treatment of idiopathic scoliosis
FTRB: Full time rigid bracing
PSSE: Physiotherapeutic scoliosis-specific exercises
RAB: Rotational angular breathing
SOSORT: Society on scoliosis orthopaedic and rehabilitation treatment

## References

[1] Negrini S, Aulisa AG, Aulisa L, Circo AB, de Mauroy JC, Durmala J (2012). 2011 SOSORT guidelines: Orthopaedic and Rehabilitation treatment of idiopathic scoliosis during growth. Scoliosis.

[2] Bettany-Saltikov J, Parent E, Romano M, Villagrasa M, Negrini S (2014). Physiotherapeutic scoliosis-specific exercises for adolescents with idiopathic scoliosis. Eur J Phys Rehabil Med.

[3] Lehnert-Schroth C (2000). Three-dimensional Scoliosis Treatment - The Schroth Orthopedic Breathing System [Dreidimensionale Skoliosebehandlung: eine physiotherapeutische Spezialmethode zur Verbesserung von Rückgratverkrümmungen: Atmungs-Orthopädie-System Schroth].

[4] Negrini S, Atanasio S, Fusco C, Zaina F (2009). Effectiveness of complete conservative treatment for adolescent idiopathic scoliosis (bracing and exercises) based on SOSORT management criteria: results according to the SRS criteria for bracing studies - SOSORT Award 2009 Winner. Scoliosis.

[5] Anwer S, Alghadir A, Abu Shaphe M, Anwar D (2015). Effects of exercise on spinal deformities and quality of life in patients with adolescent idiopathic scoliosis. Biomed Res Int.

[6] Negrini S, Fusco C, Minozzi S, Atanasio S, Zaina F, Romano M (2008). Exercises reduce the progression rate of adolescent idiopathic scoliosis: results of a comprehensive systematic review of the literature. Disabil Rehabil.

[7] Schreiber S, Parent EC, Moez EK, Hedden DM, Hill D, Moreau MJ (2015). The effect of Schroth exercises added to the standard of care on the quality of life and muscle endurance in adolescents with idiopathic scoliosis-an assessor and statistician blinded randomized controlled trial: “SOSORT 2015 Award Winner”. Scoliosis.

[8] Kuru T, Yeldan I, Dereli EE, Ozdincler AR, Dikici F, Colak I (2016). The efficacy of three-dimensional Schroth exercises in adolescent idiopathic scoliosis: a randomised controlled clinical trial. Clin Rehabil.

[9] Lohse K, Shirzad N, Verster A, Hodges N, Van der Loos HF (2013). Video games and rehabilitation: using design principles to enhance engagement in physical therapy. J Neurol Phys Ther.

[10] Saposnik G, Levin M, Outcome Research Canada (SORCan) Working Group (2011). Virtual reality in stroke rehabilitation: a meta-analysis and implications for clinicians. Stroke.

[11] Taylor M, Griffin M (2015). The use of gaming technology for rehabilitation in people with multiple sclerosis. Mult Scler.

[12] Maddison R, Foley L, Ni Mhurchu C, Jiang Y, Jull A, Prapavessis H (2011). Effects of active video games on body composition: a randomized controlled trial. Am J Clin Nutr.

[13] Trost SG, Sundal D, Foster GD, Lent MR, Vojta D (2014). Effects of a pediatric weight management program with and without active video games a randomized trial. JAMA Pediatr.

[14] Lehnert-Schroth C, Gröbl P. Three-dimensional Scoliosis Treatment; the Schroth Orthopedic Breathing System [Dreidimensionale Skoliosebehandlung; Atmungs-Orthopädie System Schroth]. 8th ed. München: Urban & Fischer; 2014.

[15] Shumway-Cook A, Woollacott M (2006). H. Motor-Control: Translating Research into Clinical Practice. 3rd ed.

[16] Zwicker JG, Harris SR (2009). A reflection on motor learning theory in pediatric occupational therapy practice. Can J Occup Ther.

[17] Sullivan KJ, Kantak SS, Burtner PA (2008). Motor learning in children: feedback effects on skill acquisition. Phys Ther.

[18] Chiviacowsky S, Wulf G, de Medeiros FL, Kaefer A, Tani G (2008). Learning benefits of self-controlled knowledge of results in 10-year-old children. Res Q Exerc Sport.

[19] Huber M (2008). Feedback as therapeutical technique - sometimes less is more [Feedback als therapeutische Technik - weniger ist manchmal mehr]. Ergopraxis.

[20] Carman DL, Browne RH, Birch JG (1990). Measurement of scoliosis and kyphosis radiographs. Intraobserver and interobserver variation. J Bone Joint Surg Am.

[21] Gstoettner M, Sekyra K, Walochnik N, Winter P, Wachter R, Bach CM (2007). Inter- and intraobserver reliability assessment of the Cobb angle: manual versus digital measurement tools. Eur Spine J.

[22] Schreiber S, Parent EC, Watkins EM, Hedden DM (2012). An algorithm for determining scoliosis curve type according to Schroth. Scoliosis.

